# Buprenorphine and Methadone as Opioid Maintenance Treatments for Heroin-Addicted Patients Induce Oxidative Stress in Blood

**DOI:** 10.1155/2019/9417048

**Published:** 2019-04-09

**Authors:** Christonikos Leventelis, Nikolaos Goutzourelas, Aikaterini Kortsinidou, Ypatios Spanidis, Georgia Toulia, Antzouletta Kampitsi, Christina Tsitsimpikou, Dimitrios Stagos, Aristidis S. Veskoukis, Demetrios Kouretas

**Affiliations:** ^1^Department of Biochemistry and Biotechnology, University of Thessaly, 41500 Larissa, Greece; ^2^Organization Against Drugs, 10433 Athens, Greece; ^3^Department of Nursing, University of West Attica, 12243 Athens, Greece; ^4^General Anticancer Hospital “Agios Savvas”, 11522 Athens, Greece; ^5^General Chemical State Laboratory of Greece, 11521 Athens, Greece

## Abstract

Buprenorphine and methadone are two substances widely used in the substitution treatment of patients who are addicted to opioids. Although it is known that they partly act efficiently towards this direction, there is no evidence regarding their effects on the redox status of patients, a mechanism that could potentially improve their action. Therefore, the aim of the present investigation was to examine the impact of buprenorphine and methadone, which are administered as substitutes to heroin-dependent patients on specific redox biomarkers in the blood. From the results obtained, both the buprenorphine (*n* = 21) and the methadone (*n* = 21) groups exhibited oxidative stress and compromised antioxidant defence. This was evident by the decreased glutathione (GSH) concentration and catalase activity in erythrocytes and the increased concentrations of thiobarbituric acid reactive substances (TBARS) and protein carbonyls in the plasma, while there was no significant alteration of plasma total antioxidant capacity (TAC) compared to the healthy individuals (*n* = 29). Furthermore, methadone revealed more severe oxidant action compared to buprenorphine. Based on relevant studies, the tested substitutes mitigate the detrimental effects of heroin on patient redox status; still it appears that they need to be boosted. Therefore, concomitant antioxidant administration could potentially enhance their beneficial action, and most probably, buprenorphine that did not induce oxidative stress in such a severe mode as methadone, on the regulation of blood redox status.

## 1. Introduction

Drug addiction is a serious health problem that modern society has to face. It is indicative that mortality rates due to the increasing prevalence of opioid use have risen approaching an epidemic scale in some countries [1]. According to recent epidemiological data, there is an upward trend in Europe with regard to the number of overdose deaths, and intriguingly, opioids are responsible for the 81% of them [2]. In addition, it has been reported that in the European Union, opioids are the main substances of use (i.e., 38% of all cases), whereas heroin comprises the 79% of them. There is also a serious issue of this kind in North America since there has been observed enhanced morbidity and mortality associated with the abuse of prescription opioids, heroin, and lately, the use of high-potency synthetic opioids, especially fentanyl derivatives [2].

Opioids in general have twofold inhibitory action. They act both at the presynaptic nerve terminal by inhibiting neurotransmitter release and at the postsynaptic neuron. Specifically, they primarily block *μ* (mu) receptors, thus, preventing the secretion of *γ*-aminobutyric acid (GABA) that acts on dopaminergic neurons by inhibiting dopamine release. The inhibitory action of opioids on GABA results in increased dopamine release by dopaminergic neurons in the ventral tegmental area (VTA), which is associated with the reward system through projecting to the nucleus accumbens [3]. The latter are considered the neural mediators for food intake, sexual behavior, motivation for reward, stress-related behavior, and substance dependence [4, 5]. Some of the well-described (side) effects of opioid use are analgesia, respiratory depression, euphoria, and psychological dependence [6]. The augmentation of dopamine release appears to be responsible for addiction in opioids. Interestingly, the increase of dopamine induced by stimuli associated with pleasure that are an outcome of opioid substance use leads to memorizing signals announcing the reward. Therefore, when the dopamine system is overstimulated, the desire to repeat this experience may be at the expense of other important targets [7, 8].

There are several studies in the literature demonstrating a connection between addiction in opioids and oxidative stress in neuron cells. Noteworthy, repeated use of large opioid doses causes permanent damage to the dopamine mechanism. This is due to elevated dopamine release, hence, causing its autooxidation that generates 3,4-dihydroxyphenylacetic acid (DOPAC), a metabolite of dopamine and H_2_O_2_ [9–11]. Hydrogen peroxide can subsequently react with metal ions (Fe^++^ and Cu^+^) and during the Fenton reaction generates OH^·^ radical, which is probably the most reactive free radical in the cellular environment potentially inducing oxidative stress [12–18]. Furthermore, it has been shown that the increased dopamine release through its oxidation leads to the production of quinone radicals lowering the GSH : GSSG ratio and, therefore, the available reductive equivalents [17, 19–21]. The reactive species induced by opioid use activate the Jun N-terminal kinase/stress-activated protein kinase pathway (JNP/SAPK) causing neuron cell apoptosis [22, 23]. Due to potentially high levels of dopamine oxidation, it has been hypothesized that dopaminergic neuron endings more likely maintain dopamine levels in synaptic vesicles than neutralize the dopamine oxidation resulting in neurotoxic effects [10, 24].

Buprenorphine and methadone treatment is a common practice for rehab of individuals that use addictive substances. It involves the prescription of these drugs as substitutes to the opioids that a patient is dependent on [25–28]. Buprenorphine seems to be more effective than methadone because it causes less analgesia since it is not a full agonist of *μ* receptors [29]. It has been also demonstrated that methadone reduces opioid tolerance and alters redox status, thus alleviating the side effects of opioids [30]. To this end, there are numerous substitution programs worldwide administering buprenorphine and methadone and it has been reported that they increase the probability of recovery for the addictive individuals [31].

To our knowledge, there is scarce evidence that methadone and buprenorphine act through redox-related mechanisms [32, 33]. However, the literature lacks observational studies regarding their effects on the redox status of individuals that are addictive to opioid substances. It is known that drug addictions have a negative impact on the systemic antioxidant defences. Therefore, the goal of the present investigation was to examine the effects of methadone and buprenorphine, when used as substitute treatments, on the redox status of patients suffering from heroin addiction.

## 2. Materials and Methods

### 2.1. Participants

Seventy-one subjects participated in the present investigation. They were randomly divided into two groups, namely, the observation group (*n* = 42), which includes patients being under opioid maintenance treatment (OMT) in the therapeutic units of Attica Organization Against Drugs in Greece, and the control group (*n* = 29) comprising healthy individuals without prior contact with substances able to induce addiction. The OMT group was further divided in the MMT (methadone maintenance treatment) (*n* = 21) and the BMT (buprenorphine maintenance treatment) (*n* = 21) groups. The participating patients were fully informed about the purpose and objectives of the study. All necessary information and safeguards were provided to ensure the confidentiality of data, and each patient signed a consensus form before the study began. According to our inclusion criteria, all subjects were over 20 years of age and were long-term heroin or other opioid drug users and suffering from physical and mental dependence due to using. Participants with severe psychopathology and other serious medical problems, such as infection by human immunodeficiency virus (HIV) or hepatitis B virus (HBC), were excluded from the study. Patients with relapse to other addictive substances were not also included. In order to avoid this, all participants underwent weekly urine tests during the three-month period of the substitution treatment (i.e., methadone or buprenorphine) to rule out the use of other substances (i.e., opioids, methamphetamine, methadone, benzodiazepines, cannabis, tetrahydrocannabinol, amphetamine, and buprenorphine by one-step multidrug test kits). All subjects were found negative for substance abuse. The subjects' demographic data of the participants in the OMT programs including age, gender, area of residence, years attending Organization Against Drugs (OKANA, Athens, Greece) programs, age started using, and duration of using addictive substances before the OMT were obtained. All applied experimental procedures were in line with the European Union Guidelines laid down in the 1964 Declaration of Helsinki and approved by the Institutional Review Board of the University of Thessaly (Larissa, Greece) and the Organization Against Drugs (Athens, Greece).

### 2.2. Drug Administration

Commercial methadone hydrochloride solution (10 mg/ml) and buprenorphine/buprenorphine-naloxone pills (2-8 mg) were used for regular doses. The mean daily dose of methadone was 60 mg. According to the relevant literature, methadone doses of 40-50 mg or 80-100 mg per day are effective as opioid maintenance treatments for heroin-addicted patients [34, 35]. However, given that, to our knowledge, there are no studies reporting an optimal methadone daily dose, and additionally, the inter-individual differences of patients constitute a crucial factor for drug efficiency; a medium dose (i.e., 60 mg) was chosen to be administered. With respect to buprenorphine, the mean daily dose was 16 mg. On the basis of the available data, this dosage regimen is the most commonly used in order buprenorphine to exert its action [35]. The substitutes were administered to the patients for three months that, according to previous studies, this is a proper time period for exerting their action without any side effects, although no relevant publications exist regarding their effects on blood redox status. It is worth mentioning that the patients used only heroin before the three-month period of the experimental procedure, whereas, as stated above, they received no other substances during it.

### 2.3. Blood Collection and Handling

Blood samples were drawn from a forearm vein of seated individuals and stored in ethylenediaminetetraacetic acid (EDTA; Becton-Dickinson, Franklin Lakes, NJ, USA) tubes. The samples were immediately centrifuged (1,370 g, 10 min, 4°C), and the supernatant (i.e., plasma) was collected for the measurement of the concentrations of protein carbonyls (PC) as a biomarker of protein oxidation, thiobarbituric acid reactive substances (TBARS) as a biomarker of lipid peroxidation, and total antioxidant capacity (TAC) as a crude biomarker for assessing blood antioxidant potency [36]. Subsequently, distilled water (dH_2_O, 1 : 1 (*v*/*v*)) was added to the packed erythrocytes; they were inverted vigorously and centrifuged (4,020 g, 15 min, 4°C). The supernatant (i.e., the erythrocyte lysate) was collected and used for measuring the activity of catalase (CAT) as a fundamental antioxidant enzyme. A small amount of erythrocyte lysate (i.e., 500 *μ*l) was treated with 5% trichloroacetic acid (TCA, Sigma-Aldrich, Munich, Germany) (1 : 1 (*ν*/*ν*)), vortexed, and centrifuged (28,000 g, 5 min, 4°C). The supernatant was then removed, and the same procedure was repeated once more. Τhe clear supernatant was transferred in plastic tubes and used for the measurement of reduced glutathione (GSH) concentration as the most potent intrinsic antioxidant molecule [36]. Plasma and erythrocyte lysates were stored at -80°C until further analysis.

### 2.4. Protocols for the Measurement of Redox Biomarkers

The concentration of PC was determined on the basis of the method of Patsoukis et al. [37] as described by Veskoukis et al. [38]. In this assay, 50 *μ*l of 20% TCA was added to 50 *μ*l of plasma; the mixture was incubated for 15 min in an ice bath and centrifuged (15,000 g, 5 min, 4°C). The supernatant was removed, and 500 *μ*l of 10 mM 2,4-dinitrophenylhydrazine (DNPH; Sigma-Aldrich, Munich, Germany) (in 2.5 N HCl) for the samples and 500 *μ*l of 2.5 N HCl for the blank were added to the pellet. The samples were incubated in the dark at room temperature (RT) for 1 h with intermittent vortexing every 15 min and were centrifuged (15,000 g, 5 min, 4°C). The supernatant was removed, and 1 ml of 10% TCA was added; the samples were vortexed and centrifuged (15,000 g, 5 min, 4°C). The supernatant was then discarded, and 1 ml of ethanol-ethyl acetate mixture (1 : 1 (*v*/*v*)) was added; the samples were vortexed and centrifuged (15,000 g, 5 min, 4°C). This washing step was repeated twice. The supernatant was discarded again, and 1 ml of 5 M urea (pH = 2.3) was added; the samples were vortexed and incubated at 37°C for 15 min. They were then centrifuged (15,000 g, 3 min, 4°C), and the absorbance was monitored at 375 nm using a spectrophotometer (Hitachi U-1900; serial no. 2023-029; Hitachi, Tokyo, Japan). The determination of PC concentration was based on the millimolar extinction coefficient of DNPH (22 l/mmol/cm).

The assay for the determination of TBARS concentration was based on the method described by Keles et al. [39]. According to it, 100 *μ*l of plasma (or dH_2_O for the blank) was mixed with 500 *μ*l of 35% TCA (Merck KGaA, Darmstadt, Germany) and 500 *μ*l of Tris-HCl (Sigma-Aldrich, St. Louis, MO, USA; 200 mM, pH = 7.4) and incubated at RT for 10 min. One milliliter of 2 M sodium sulfate (Na_2_SO_4_) and 55 mM of thiobarbituric acid solution were added, and the samples were incubated in a waterbath at 95°C for 45 min. The samples were cooled on ice and vortexed following the addition of 1 ml of 70% TCA. Then, they were centrifuged (15,000 g, 3 min, 25°C) and the absorbance of the supernatant was monitored at 530 nm. The calculation of the TBARS concentration was based on the molar extinction coefficient of malondialdehyde [40].

TAC measurement was based on the method described by Janaszewska and Bartosz [41]. Briefly, 20 *μ*l of plasma (or dH_2_O for the blank) was added to 480 *μ*l of sodium potassium phosphate buffer (10 mM, pH  = 7.4) and 500 *μ*l of 0.1 mM 1,1-diphenyl-1-picrylhydrazyl radical (DPPH^·^) and the samples were incubated in the dark at RT for 60 min. The samples were then centrifuged (20,000 g, 3 min, 25°C), and the absorbance was monitored at 520 nm. TAC determination was based on the mmol of DPPH^·^ reduced by the antioxidants present in the plasma [40].

CAT activity was determined on the basis of the method of Aebi [42] as described by Veskoukis et al. [38]. In particular, 4 *μ*l οf erythrocyte lysate (diluted 1 : 10) was added to 2,991 *μ*l οf sodium potassium phosphate buffer (67 mM, pH = 7.4) and the samples were incubated at 37°C for 10 min. Then, 5 *μ*l of 30% H_2_O_2_ solution was added and the change in absorbance was immediately read at 240 nm for 130 sec. The calculation of CAT activity was based on the molar extinction coefficient of H_2_O_2_.

GSH concentration was measured based on the method of Reddy et al. [43] as described by Veskoukis et al. [38]. For this assay, 20 *μ*l of erythrocyte lysate (or dH_2_O for the blank) previously treated with 5% TCA was mixed with 660 *μ*l of sodium-potassium phosphate buffer (67 mM, pH = 8) and 330 *μ*l of 1 mM 5,5-dithiobis-2 nitrobenzoate (DTNB; Sigma-Aldrich, Munich, Germany). The samples were incubated in the dark at RT for 45 min, and the absorbance was monitored at 412 nm. The GSH concentration was calculated on the basis of a standard curve using commercially available standards.

Total protein in plasma samples was measured using Bradford reagent. Hemoglobin concentration in erythrocyte lysates was determined with a commercially available kit (Drabkin) according to the manufacturer's instructions. Each assay was performed in triplicate at two different occasions.

### 2.5. Statistical Analysis

Data regarding redox biomarkers were analyzed by one-way ANOVA followed by Dunnett's test for multiple pairwise comparisons. Correlation analysis between redox biomarkers and demographic data was carried out using Pearson's correlation coefficient. The statistical significance was set at *p* < 0.05. For all statistical analyses, SPSS software version 21.0 (SPSS Inc., Chicago, IL, USA) was used. Data are presented as the mean ± standard error of the mean (SEM).

## 3. Results

### 3.1. Demographic Data

With respect to the control group, the mean age was 36.3 ± 3.2 years old and 68.9% of the participants were men. The demographic data of the participants in the OMT programs are depicted in Table 1. In brief, the mean age was 40.5 ± 1.3 years old, 68.9% of the participants were men, they mostly lived in urban areas (i.e., 92.9%), the mean time of attending OKANA programs was 0.98 ± 0.17 years, and they mainly started using addictive substances at the age of 11-20 (i.e., 66.7%) for 11-20 years (i.e., 45.2%). Furthermore, Spearman's correlations between the demographic data and the redox biomarkers exerted no statistical significance.

### 3.2. Redox Biomarkers

**Figure 1:**
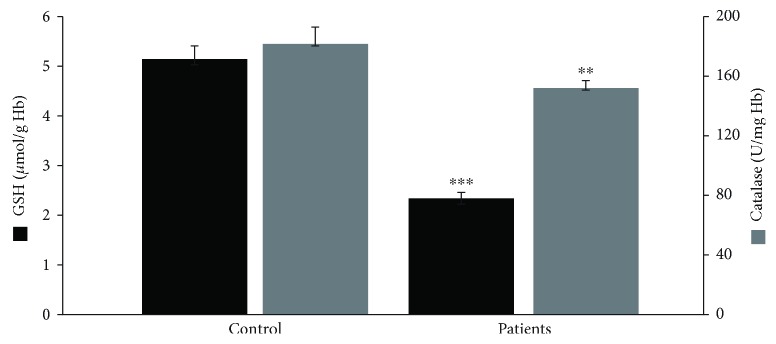
GSH concentration and catalase activity in the control group (*n* = 29) and the OMT patients as a whole (*n* = 42). ^∗∗^^,^^∗∗∗^Significantly different compared to the control group (*p* < 0.05 and *p* < 0.001, respectively).

**Figure 2:**
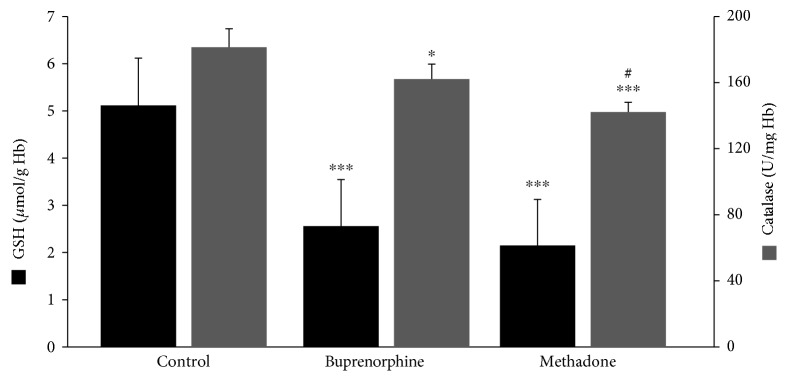
GSH concentration and catalase activity in the control (*n* = 29), the BMT (buprenorphine) (*n* = 21), and the MMT (methadone) (*n* = 21) groups. ^∗^^,^^∗∗∗^Significantly different compared to the control group (*p* < 0.05 and *p* < 0.001, respectively). ^#^Significantly different compared to the buprenorphine group (*p* < 0.05).

**Figure 3:**
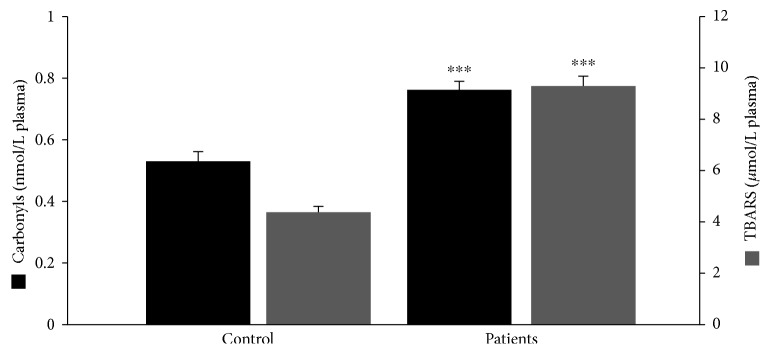
Protein carbonyl and TBARS concentrations in the control group (*n* = 29) and the OMT patients as a whole (*n* = 42). ^∗∗∗^Significantly different compared to the control group (*p* < 0.001).

**Figure 4:**
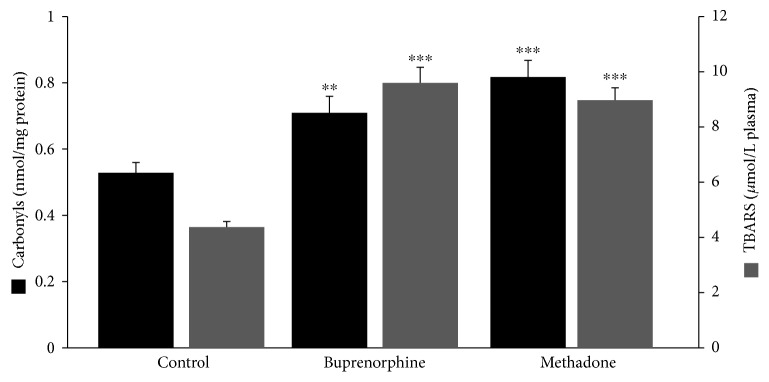
Protein carbonyl and TBARS concentrations in the control (*n* = 29), the BMT (buprenorphine) (*n* = 21), and the MMT (methadone) (*n* = 21) groups. ^∗∗^^,^^∗∗∗^Significantly different compared to the control group (*p* < 0.01 and *p* < 0.001, respectively).

**Figure 5:**
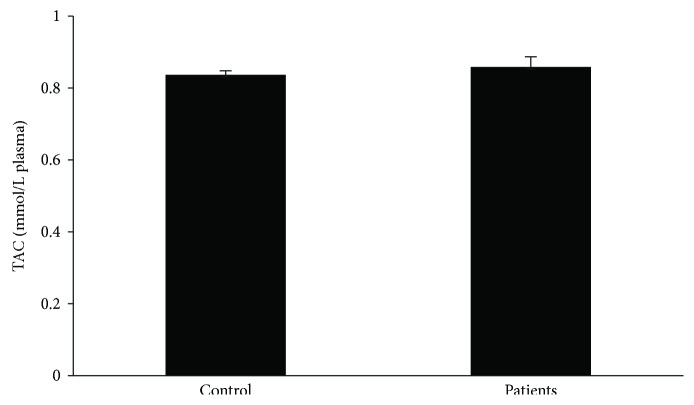
TAC levels in the control group (*n* = 29) and the OMT patients as a whole (*n* = 42).

**Figure 6:**
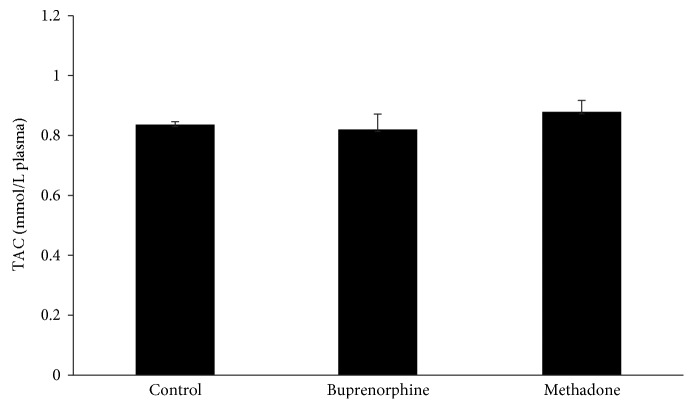
TAC levels in the control (*n* = 29), the BMT (buprenorphine), (*n* = 21) and the MMT (methadone) (*n* = 21) groups.

Regarding GSH concentration and catalase activity, they were both found significantly decreased in the group of patients as a whole compared to the control by 54% and 16%, respectively (Figure 1). According to the results on the basis of each administered substance, GSH concentration was reduced in both the BMT and MMT groups compared to the control by 51% and 58%, respectively (Figure 2). No significant difference between the BMT and MMT groups was observed. Furthermore, CAT activity decreased in both the BMT and MMT groups compared to the control by 10% and 22%, respectively. There was also a significant difference in CAT activity between the MMT and BMT groups (Figure 2). With respect to PC and TBARS concentrations, a significant increase was observed in the levels of both biomarkers in the group of patients as a whole compared to the control by 34% and 112%, respectively (Figure 3). On the basis of each administered substance, PC concentration was increased in both the BMT and MMT groups by 34% and 51%, respectively (Figure 4). TBARS concentration was increased in both the BMT and MMT groups compared to the control by 120% and 105%, respectively. No significant differences between the BMT and MMT groups in either PC or TBARS levels were noticed. Finally, there were no alterations in TAC levels between the tested groups of participants (Figures 5 and 6).

## 4. Discussion

The main findings of the present investigation indicate that buprenorphine and methadone, two opioid substitutes administered to heroin users in order to get into the rehab period, induce oxidative stress compared to healthy individuals. It becomes apparent, hence that although they impair the unpleasant and often inhumane side effects of heroin, they still disrupt redox balance in the blood of patients.

It is worth mentioning that methadone is a full agonist of the *μ*-opioid receptor, whereas buprenorphine is a partial agonist of the *μ*-opioid receptor and a *κ*-receptor antagonist [44, 45]. Both agents are used in substitution treatment to reduce opioid damage, which is referred to as MMT (methadone maintenance treatment) and BMT (buprenorphine maintenance treatment), respectively [46, 47]. Several studies have asserted the relation of opioid cytotoxic effects with the disruption of redox balance and have stressed the protective role of GSH. Specifically, increased production of reactive oxygen species (ROS) has been associated with heroin-induced intracellular dopamine and DOPAC [16], whereas dopamine infusion in GSH-depleted SK-N-SH neuroblastoma cells increased apoptosis, nuclear DNA fragmentation, and cell membrane lesions [48]. In line with the above studies, it has also been demonstrated that a reduction of extracellular GSH was observed when astrocytes were cultured in a dopamine-rich solution indicating that dopamine is an oxidant agent especially in the absence of GSH [20], while methamphetamine-treated rats exhibited reduced GSH concentration in the striatum [21].

The negative impact of both opioids used in the present investigation (i.e., buprenorphine and methadone) on blood antioxidant status is depicted by the results in GSH concentration and CAT activation. Specifically, they were both found reduced at the patient group compared to the healthy individuals implying that their antioxidant defence was compromised. In addition, methadone seems to have a more severe effect as indicated by the significantly lower values of catalase compared to buprenorphine. These results are supported by previous findings [49–52]. Specifically, methadone appears to have a greater impact on lowering antioxidant defence since patients under MMT have shown a depletion of GSH and CAT levels [50, 52]. Nevertheless, studies comparing heroin users and methadone-treated patients have reported that the MMT group exhibits improvement in redox biomarkers [30, 53, 54]. On the same grounds, TAC is decreased after opioid administration, especially heroin [9, 55–57], but it seems that substitutes have a positive effect [9, 58] increasing the antioxidant capacity of the organism. These findings are optimistic because they support the notion that although substitute treatment induces oxidative stress, this occurs in much less extent in comparison to heroin, thus reinforcing its use at rehab programs. In addition, reduction of heroin damage by substitute treatment enhances total antioxidant activity [58]. With respect to the biomarkers illustrating the oxidative modification of biomolecules, both PC and TBARS concentrations were elevated in the MMT and BMT groups. Several studies are in line with our findings indicating that methadone and buprenorphine induce oxidative stress [55, 59]. Indeed, malondialdehyde (MDA) levels were found elevated in opioid addicts [60] and in heroin users compared to methadone-administered individuals [9, 32]. Similarly, an increase in TBARS concentration has been observed in mice following heroin administration [56], whereas no significant difference after the administration of buprenorphine in comparison to the healthy animals was observed in rats [51].

It is known that, although buprenorphine and methadone induce oxidative stress as is the case in the present study too, they do it in lower extent in comparison to opioids, such as heroin that an individual can potentially be dependent on. Comparing the two agonists, buprenorphine appears to have a less severe impact on oxidative stress keeping a higher burden of intrinsic antioxidants, a fact that is in conformity with previous results reporting that buprenorphine inhibits oxidative stress [61]. To this direction, it has been proposed that internalizing opioids (methadone, fentanyl, sufentanil) activate phospholipase D2 (PLD2) and lead to enhanced ROS generation, while noninternalizing agonists (i.e., buprenorphine) do not [62]. PLD2 activation is contributed to the endocytosis of the *μ*-receptor and the development of opioid tolerance [63, 64]. PLD2 is considered to play an important role not only in the membrane trafficking of the *μ* receptor but also in the functional selectivity of opioids at it. Furthermore, the increase of free radical generation by PLD2-activating opioids is also implicated in other signaling pathways induced by growth factors playing an important role in cell proliferation and differentiation [60]. The mechanism by which ROS mediate cell proliferation appears to be associated with the activation of extracellular signal-regulated kinase 5 and p38 MAPK, which are redox-sensitive [65]. Furthermore, the product of PLD, phosphatidic acid, has been found to lead through its interaction with the mammalian target of rapamycin (mTOR) in the release and activation of cytokines [66, 67]. Notably, it has been demonstrated that exogenous administration of antioxidants can act protectively against free radical generation induced by opioids used for maintenance treatment [32, 56].

## 5. Conclusion

The present investigation asserts that buprenorphine and methadone, two widely used substitutes for opioid maintenance treatment, induce oxidative stress and compromise blood antioxidant defence mechanisms. It is noteworthy that according to the findings of other relevant studies, they (especially buprenorphine) attenuate the severe oxidative impact of heroin and other opioids that cause addiction. Thus, with respect to improving the antioxidant burden of patients dependent on opioids, it appears that buprenorphine and methadone act towards the desirable direction. However, as it has been previously reported, concomitant antioxidant administration could potentially enhance their beneficial action by regulating blood redox status.

## Figures and Tables

**Table 1:** Demographic data of the participants in the OMT programs (*n* = 42).

Age (yrs)	40.5 ± 1.3
Gender (%)	Men **(73.8)**
	Women **(26.2)**
Area of residence (%)	Urban **(92.9)**
	Rural **(7.1)**
Years attending OKANA programs	0.98 ± 0.17
Age started using addictive substances (%)	11-20 **(66.7)**
	21-30 **(26.2)**
	31-40 **(7.1)**
Years using addictive substances (%)	0-10 **(21.4)**
	11-20 **(45.2)**
	21-30 **(21.4)**
	31-40 **(11.9)**

## Data Availability

The data used to support the findings of this study are available from the corresponding author upon request.
